# Supplementary material for: Comparing 3D Tooth Movement When Implementing the Same Virtual Setup on Different Software Packages
Source: J Clin Med. 2022 Sep 12;11(18):5351. doi: 10.3390/jcm11185351 (PMC9503059; doi:10.3390/jcm11185351)
Supplement: Supplementary file 1 [file jcm-11-05351-s001.zip › jcm-1867811-supplementary.pdf]

Journal of
Clinical Medicine

MDPI

Supplementary Materials

Comparing 3D Tooth Movement When Implementing the Same Virtual Setup on Different Software Packages

Supplementary Table S1. Geomagic® Control X Superimpositions.

	Absolute Average (mm)	Standard Deviation (mm)	(+) Average (mm)	(-) Average (mm)
Lower SureSmile® 001	0.0044	0.1289	0.0625	-0.0479
Upper SureSmile® 001	-0.0028	0.0659	0.0399	-0.0477
Lower Ortho Insight 001	0.0131	0.0893	0.066	-0.0594
Upper Ortho Insight 001	0.0112	0.0636	0.0255	-0.0423
Lower 3Shape 001	0.0206	0.0881	0.0672	-0.0438
Upper 3Shape 001	0.0137	0.0985	0.0832	-0.0663
Lower SureSmile® 002	-0.0017	0.07	0.0446	-0.0519
Upper SureSmile® 002	-0.0007	0.0837	0.0553	-0.0626
Lower Ortho Insight 002	0.0222	0.079	0.0696	-0.0476
Upper Ortho Insight 002	0.0159	0.0642	0.0556	-0.0399
Lower 3Shape 002	0.0302	0.1115	0.0975	-0.0787
Upper 3Shape 002	0.0202	0.0994	0.0837	-0.0668
Lower SureSmile® 003	0.0146	0.1161	0.0645	-0.0435
Upper SureSmile® 003	-0.0053	0.0936	0.051	-0.0617
Lower Ortho Insight 003	0.0085	0.0724	0.0551	-0.0459
Upper Ortho Insight 003	0.0048	0.0893	0.0588	-0.0603
Lower 3Shape 003	0.0226	0.135	0.1053	-0.0927
Upper 3Shape 003	0.0078	0.1163	0.0862	-0.0821
Lower SureSmile® 004	0.0144	0.1514	0.061	-0.0454
Upper SureSmile® 004	0.0092	0.0618	0.0485	-0.0433
Lower Ortho Insight 004	0.0117	0.0695	0.0579	-0.0427
Upper Ortho Insight 004	0.0102	0.0711	0.0555	-0.493
Lower 3Shape 004	0.0012	0.1064	0.0868	-0.0832
Upper 3Shape 004	0.0155	0.0986	0.0853	-0.0723
Lower SureSmile® 005	-0.008	0.0597	0.0414	-0.0482
Upper SureSmile® 005	-0.0005	0.0661	0.0423	-0.0463
Lower Ortho Insight 005	0.007	0.0942	0.0653	-0.061
Upper Ortho Insight 005	0.013	0.0829	0.0649	-0.0527
Lower 3Shape 005	0.0095	0.1113	0.0904	-0.0796
Upper 3Shape 005	0.0219	0.1043	0.0862	-0.0629
Lower SureSmile® 006	0.0017	0.0555	0.0382	-0.0323
Upper SureSmile® 006	0.0027	0.0735	0.0515	-0.0433
Lower Ortho Insight 006	0.0178	0.0643	0.0541	-0.029
Upper Ortho Insight 006	0.0112	0.054	0.0415	-0.0276
Lower 3Shape 006	0.0237	0.079	0.0696	-0.0448
Upper 3Shape 006	0.0099	0.0757	0.0629	-0.0425
Lower SureSmile® 007	0.0007	0.0601	0.0415	-0.0426
Upper SureSmile® 007	0.0039	0.0767	0.0443	-0.0406
Lower Ortho Insight 007	0.0071	0.0813	0.0546	-0.0533
Upper Ortho Insight 007	0.0135	0.0763	0.0571	-0.0434
Lower 3Shape 007	0.0261	0.1126	0.0993	-0.0673

Upper 3Shape 007	0.0219	0.0945	0.0781	-0.0601
Lower SureSmile® 008	0.0033	0.074	0.0536	-0.0572
Upper SureSmile® 008	-0.0002	0.0626	0.0426	-0.0437
Lower Ortho Insight 008	0.0131	0.0861	0.0692	-0.0465
Upper Ortho Insight 008	0.0182	0.0866	0.0624	-0.0542
Lower 3Shape 008	0.0197	0.1047	0.0751	-0.0552
Upper 3Shape 008	0.0205	0.0863	0.0751	-0.0559
Lower SureSmile® 009	0.0124	0.0566	0.0453	-0.0366
Upper SureSmile® 009	-0.0006	0.062	0.0487	-0.0413
Lower Ortho Insight 009	0.0159	0.064	0.0535	-0.0372
Upper Ortho Insight 009	0.0128	0.0748	0.0573	-0.0442
Lower 3Shape 009	0.0201	0.0893	0.0782	-0.0563
Upper 3Shape 009	0.0148	0.0742	0.0615	-0.046
Lower SureSmile® 010	-0.0011	0.0598	0.0383	-0.0399
Upper SureSmile® 010	-0.0012	0.0738	0.0428	-0.0428
Lower Ortho Insight 010	0.0077	0.0846	0.0602	-0.0587
Upper Ortho Insight 010	0.0191	0.0737	0.0631	-0.0453
Lower 3Shape 010	0.0236	0.0919	0.0735	-0.055
Upper 3Shape 010	0.0241	0.0945	0.081	-0.0654
Lower SureSmile® 011	-0.0048	0.0714	0.0519	-0.0552
Upper SureSmile® 011	0.0048	0.0989	0.0542	-0.0488
Lower Ortho Insight 011	0.0032	0.0818	0.06	-0.0601
Upper Ortho Insight 011	0.0009	0.0997	0.0679	-0.0619
Lower 3Shape 011	0.0198	0.0905	0.0737	-0.0516
Upper 3Shape 011	0.03	0.097	0.0839	-0.0589
Lower SureSmile® 012	0.0056	0.0885	0.0523	-0.0451
Upper SureSmile® 012	0.0055	0.0784	0.0502	-0.0455
Lower Ortho Insight 012	0.013	0.0661	0.0535	-0.0417
Upper Ortho Insight 012	0.0101	0.08	0.0545	-0.0509
Lower 3Shape 012	0.0224	0.0925	0.0762	-0.0589
Upper 3Shape 012	0.0216	0.105	0.086	-0.0537
Lower SureSmile® 013	0.1609	0.485	0.321	-0.0723
Upper SureSmile® 013	0.0141	0.0696	0.0589	-0.0478
Lower Ortho Insight 013	0.0028	0.0733	0.0549	-0.0521
Upper Ortho Insight 013	0.009	0.0818	0.0582	-0.0506
Lower 3Shape 013	0.016	0.1057	0.0864	-0.0657
Upper 3Shape 013	0.015	0.081	0.0652	-0.0481
Lower SureSmile® 014	0.0025	0.0679	0.0506	-0.0431
Upper SureSmile® 014	0.0021	0.1516	0.0613	-0.0544
Lower Ortho Insight 014	0.0141	0.0796	0.0642	-0.0494
Upper Ortho Insight 014	0.0047	0.0869	0.0611	-0.0526
Lower 3Shape 014	0.0252	0.0853	0.0725	-0.0479
Upper 3Shape 014	0.0246	0.0865	0.0711	-0.0448
Lower SureSmile® 015	0.0008	0.062	0.0449	-0.0437
Upper SureSmile® 015	0.0032	0.0792	0.0494	-0.0462
Lower Ortho Insight 015	-0.0022	0.0958	0.0735	-0.0718
Upper Ortho Insight 015	0.0173	0.0847	0.0714	-0.055
Lower 3Shape 015	0.0079	0.0967	0.08	-0.0684
Upper 3Shape 015	0.0241	0.0819	0.0712	-0.0471

Lower SureSmile® 016	0.0063	0.073	0.0534	-0.0484
Upper SureSmile® 016	-0.0017	0.0655	0.0438	-0.0505
Lower Ortho Insight 016	0.0066	0.0712	0.0529	-0.0511
Upper Ortho Insight 016	0.0127	0.0858	0.0672	-0.0521
Lower 3Shape 016	0.0229	0.0908	0.0808	-0.062
Upper 3Shape 016	0.0357	0.0983	0.0884	-0.0578
Lower SureSmile® 017	-0.0023	0.0595	0.0384	-0.0407
Upper SureSmile® 017	-0.0055	0.0758	0.051	-0.0555
Lower Ortho Insight 017	0.0086	0.0847	0.067	-0.0535
Upper Ortho Insight 017	0.011	0.0917	0.067	-0.056
Lower 3Shape 017	0.0206	0.0978	0.0827	-0.0648
Upper 3Shape 017	0.025	0.1034	0.0814	-0.0475
Lower SureSmile® 018	-0.0035	0.0608	0.037	-0.042
Upper SureSmile® 018	-0.0033	0.0694	0.0428	-0.0487
Lower Ortho Insight 018	-0.0012	0.0771	0.0585	-0.0569
Upper Ortho Insight 018	0.0062	0.0682	0.0528	-0.0484
Lower 3Shape 018	0.0028	0.096	0.0766	-0.0726
Upper 3Shape 018	0.0177	0.0845	0.0711	-0.0468
Lower SureSmile® 019	-0.0035	0.0774	0.0505	-0.0521
Upper SureSmile® 019	0.0002	0.0667	0.0484	-0.0437
Lower Ortho Insight 019	0.003	0.0936	0.0689	-0.0627
Upper Ortho Insight 019	0.0055	0.089	0.0618	-0.0592
Lower 3Shape 019	0.0155	0.0949	0.0794	-0.0602
Upper 3Shape 019	0.0193	0.0923	0.0776	-0.0542
Lower SureSmile® 020	0.0064	0.0496	0.0407	-0.0311
Upper SureSmile® 020	0.0018	0.0605	0.0326	-0.0336
Lower Ortho Insight 020	0.0076	0.094	0.0761	-0.07
Upper Ortho Insight 020	0.0051	0.0794	0.0618	-0.0593
Lower 3Shape 020	0.0095	0.0827	0.0704	-0.0589
Upper 3Shape 020	0.013	0.0774	0.0606	-0.051
Lower SureSmile® 021	0.007	0.0794	0.0638	-0.0547
Upper SureSmile® 021	0.0047	0.1154	0.0656	-0.0641
Lower Ortho Insight 021	0.0127	0.0993	0.0749	-0.0652
Upper Ortho Insight 021	0.0124	0.0948	0.0722	-0.0608
Lower 3Shape 021	0.0164	0.0783	0.0655	-0.0501
Upper 3Shape 021	0.0227	0.0897	0.0753	-0.0565
Lower SureSmile® 022	0.0064	0.0602	0.0435	-0.0452
Upper SureSmile® 022	0.0047	0.1706	0.0623	-0.0513
Lower Ortho Insight 022	0.0143	0.062	0.0529	-0.0391
Upper Ortho Insight 022	0.0181	0.0657	0.0575	-0.0397
Lower 3Shape 022	0.019	0.0713	0.0618	-0.0413
Upper 3Shape 022	0.0143	0.0745	0.0645	-0.049
Lower SureSmile® 023	-0.01	0.0644	0.0432	-0.0507
Upper SureSmile® 023	-0.0018	0.0727	0.0451	-0.0517
Lower Ortho Insight 023	0.0046	0.0614	0.0465	-0.0432
Upper Ortho Insight 023	0.0218	0.0723	0.0598	-0.0436
Lower 3Shape 023	0.005	0.091	0.0715	-0.063
Upper 3Shape 023	0.0139	0.0806	0.0681	-0.0498
Lower SureSmile® 024	0.0017	0.0722	0.0519	-0.0568

Upper SureSmile® 024	0.0022	0.0686	0.0453	-0.0457
Lower Ortho Insight 024	0.0025	0.091	0.0684	-0.0686
Upper Ortho Insight 024	0.0079	0.0625	0.0498	-0.039
Lower 3Shape 024	0.0084	0.0875	0.0622	-0.0498
Upper 3Shape 024	0.016	0.087	0.0752	-0.0553
Lower SureSmile® 025	0.0004	0.0698	0.0434	-0.045
Upper SureSmile® 025	0.0002	0.0733	0.0433	-0.038
Lower Ortho Insight 025	0.0023	0.0907	0.0753	-0.0681
Upper Ortho Insight 025	0.0059	0.0771	0.0605	-0.0491
Lower 3Shape 025	0.0112	0.0784	0.0622	-0.0536
Upper 3Shape 025	0.0226	0.076	0.0675	-0.0418
Average of Sample	0.011	0.086	0.0637	-0.055
